# A review of visual perspective taking in autism spectrum disorder

**DOI:** 10.3389/fnhum.2013.00652

**Published:** 2013-10-08

**Authors:** Amy Pearson, Danielle Ropar, Antonia F. de C. Hamilton

**Affiliations:** ^1^Science Laboratories, Psychology Department, Durham UniversityDurham, UK; ^2^School of Psychology, University of NottinghamNottingham, UK

**Keywords:** visual perspective taking, autism spectrum disorder, spatial transformations, social cognition, spatial cognition, theory of mind

## Abstract

Impairments in social cognition are a key symptom of autism spectrum disorder (ASD). People with autism have great difficulty with understanding the beliefs and desires of other people. In recent years literature has begun to examine the link between impairments in social cognition and abilities which demand the use of spatial and social skills, such as visual perspective taking (VPT). Flavell (1977) defined two levels of perspective taking: VPT level 1 is the ability to understand that other people have a different line of sight to ourselves, whereas VPT level 2 is the understanding that two people viewing the same item from different points in space may see different things. So far, literature on whether either level of VPT is impaired or intact in autism is inconsistent. Here we review studies which have examined VPT levels 1 and 2 in people with autism with a focus on their methods. We conclude the review with an evaluation of the findings into VPT in autism and give recommendations for future research which may give a clearer insight into whether perspective taking is truly impaired in autism.

Visual perspective taking (VPT) is the ability to see the world from another person's perspective, taking into account what they see and how they see it (Flavell, 1977). In order to perform VPT successfully a person must draw upon both spatial and social information. The spatial information used in VPT includes the current position of both the viewer and the target and the position of objects in the environment in relation to the self and others (Zacks et al., 2003; Kessler and Thomson, 2009; Kessler and Wang, 2012). For instance, you are sitting at a table with a friend drinking tea, the sugar pot is on their left hand side and the teapot is oriented with the handle toward your friend. The social information used in VPT involves the simultaneous representation of two differing points of view, taking into account whether someone else can see an object, or how they see that object (Aichhorn et al., 2006). For example, your friend can see the handle of the teapot while you see the spout. By interpreting the spatial relationships between objects in a social framework it becomes possible to form a rich representation of differing viewpoints which are useful in a variety of social tasks.

Impairments in social skills are a key symptom of autism spectrum disorder (ASD) (Baron-Cohen, 1995; Happe, 1995; Frith and Frith, 2007; Frith, 2012; Senju, 2012). Research has shown that people with autism have particular difficulty with theory of mind (ToM) and representing differing beliefs (Baron-Cohen et al., 1985; Baron-Cohen, 1995; Happe, 1995; Baron-Cohen et al., 1997; Frith, 2001; Senju et al., 2009; Senju, 2012). Some theorists believe that ToM and VPT share common cognitive processes (Hamilton et al., 2009) as they both involve the simultaneous representation of two differing points of view (Aichhorn et al., 2006). If this is the case then we may expect that people with autism would be impaired at VPT as well as ToM. However, others have suggested that VPT and ToM are completely separate constructs and that it is entirely possible to be impaired at one and not the other (Leslie, 1987). Studies of whether VPT is intact in autism have been inconsistent (Reed and Peterson, 1990; Tan and Harris, 1991; Yirmiya et al., 1994; Hamilton et al., 2009). The focus of this review will be to examine studies of VPT in autism, assessing evidence for the existence of impairment. It will also consider the relationship between VPT and ToM, as well as the contribution of spatial abilities in VPT. We hope to set out a clear distinction for testing different types of VPT in autism as well as recommendations for experimental paradigms which may help to answer the question of whether these abilities may be impaired.

## Visual perspective taking

There are two different levels of VPT outlined in the literature (Flavell, 1977). VPT level one (VPT1) is the basic ability to judge what a person can and cannot see (i.e., whether an item is occluded from their line of sight). The development of VPT1 marks the period at which children begin to understand that other people may be able to see different things, for example, knowing that if a toy is behind a parent that they will not see it until they turn around. VPT1 has been measured using a variety of tasks which require children to identify whether an adult can see an item which may/may not be occluded (Masangkay et al., 1974; Flavell et al., 1981). VPT level two (VPT2) is the ability to understand that two different people viewing a scene or object simultaneously do not necessarily see objects in the same way (Flavell, 1977). Tasks measuring VPT2 require a participant to be able to say *how* someone else sees an object or scene, for example, if you are standing opposite another person looking at a car, they may see the back of the car and you may see the front.

The development of VPT skills occur in succession, with VPT1 developing first followed by VPT2 (Flavell, 1977). Currently, it is thought that VPT1 develops between the ages of 18–24 months in typical children (Flavell et al., 1981; Moll and Tomasello, 2004, 2006; Moll et al., 2007) and VPT2 later at around 4–5 years old (Gzesh and Surber, 1985). Recent advances in the field of ToM research have shown that by using more implicit measures which are less reliant on language (such as eye tracking) we can find evidence of ToM skills earlier in infancy (Southgate et al., 2007). It has also been suggested that VPT1 may be able to operate in a spontaneous and implicit fashion (Samson et al., 2005; Surtees et al., 2012). Studies of VPT to date have used only explicit measures in their methodology (i.e., asking a child to point to an item or verbally report where someone is looking). Thus, it is possible that if implicit measures similar to those of Southgate et al. (2007) were used to examine VPT we may find that it develops earlier than previously thought.

Recently, efforts have been made to provide a clear distinction between VPT levels 1 and 2, and there are several ways in which this division can be drawn. This includes reference to embodiment, implicit/explicit processing, and dyadic/triadic representations. Surtees et al. (2013) makes a distinction based on embodiment. He suggests that VPT1 tasks require only visual (line of sight) information and not an egocentric embodied transformation, while VPT2 tasks require greater spatial information processing including the full transformation of the participant's viewpoint to that of the target. A different distinction is based on implicit/explicit processing. Samson et al. (2005) suggest VPT1 can occur implicitly and spontaneously. She presented participants with images of a room in which there was a human avatar and colored disks on the walls. Participants were asked to judge how many disks they could see or how many the avatar could see. The number of disks visible to the participants and the avatar were not always the same (for example, sometimes the avatar could not see all of the disks), creating perspective congruent and perspective incongruent conditions. The authors found that typical adults' responses were slower and less accurate when the avatar's view was incongruent with their own, suggesting that they implicitly coded the avatar's visual perspective (implicit VPT1) even when not required to by the task. A third way to distinguish VPT1 and 2 focuses on the number of relationships that a participant must encode in order to perform. Warreyn et al. (2005) argue VPT1 is based upon the use of dyadic representations whereas VPT2 is reliant on triadic representations. Dyads involve a representation of the relationship between a person and an object independent of the self (i.e., Jim can see the cat). Dyadic representations appear to be based upon the use of eye gaze following and line of sight (Warreyn et al., 2005). Triadic representations, involve coding the relationship between the self, another and an object (i.e., I can see the cat's tail whereas Jim can see the cat's nose). It remains to be seen which of these three types of division between level 1 and level 2 VPT is more valuable in understanding the overall phenomenon of perspective taking.

The present review focuses on studies of VPT in autism, where these distinctions have seldom been made clear. Previous studies suggest that embodiment may be reduced in autism (Brunye et al., 2012; Kessler and Wang, 2012; Eigsti, 2013) which would imply that VPT1 should be intact but VPT2 impaired. In contrast, studies pointing to abnormal implicit ToM (in the presence of normal explicit ToM) (Senju, 2012) would predict that VPT1 should be harder in autism than VPT2. However, this might only be the case when VPT1 and 2 are tested with appropriately implicit methods, which has rarely been the case. Finally, it has been suggested that dyadic representation is intact in autism while triadic representation is impaired (Leekam et al., 1997). This implies that VPT1 should be normal in autism while VPT2 might not be. We revisit the issue of how VPT performance in autism relates to the key cognitive differences between VPT1 and VPT2 in the discussion.

**Figure 1:**
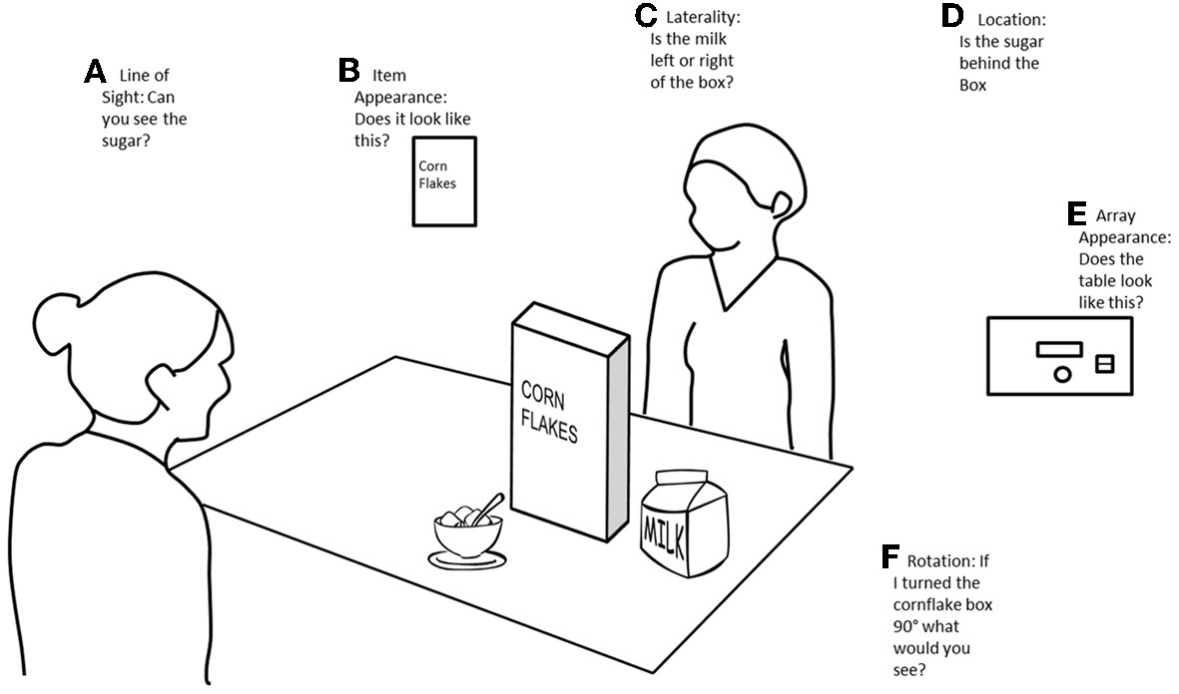
**Example of different ways in which VPT can be examined. (A)** Line of sight paradigms ask questions about whether a person can see an item, for example, “can the person on the far side of the table see the sugar bowl?” **(B)** Item appearance paradigms ask questions about how an item would appear from different points of view, for instance, “would the person on the far side of the table see the front of the cereal box?” **(C)** Laterality paradigms ask questions about the position of certain items, for instance, “is the milk to the left or right hand side of the cereal box?” **(D)** Item location paradigms ask questions about the prepositional location of items, for instance, “is the sugar bowl behind the cereal box?” **(E)** Array paradigms ask questions about the arrangement of the items in relation to each other-the way in which the array appears. For instance, participants may be shown an arrangement and asked “does the table look like this?” **(F)** Rotation paradigms ask questions about what items would look like if they were rotated to a different orientation, for instance, “if the cereal box was turned 90°, what would you see?”

One of the issues in assessing VPT in autism is the variety of methodologies that have been used. It has been suggested that people with autism may find some tasks easier to perform than others (Langdon and Coltheart, 2001) making it difficult to assert whether a lack of impairment is a result of intact VPT skills or the task used. Studies of VPT can be categorized by the types of questions they use (Figure 1). Most often studies focus on questions about item appearance (“*turn it so I can see the ___*”) or location (“*which side of the person is the counter?*”), as well as viewer or object rotations (“*imagine yourself at the blue side of the table*” vs. “*turn it so that you can see the apple*”). Studies which examine VPT1 are most likely to ask questions about line of sight (“*can this person see an object*”) rather than questions about the items appearance from different viewpoints, which is a level 2 VPT skill (Figure 1).

Evidence for intact/impaired VPT1 and VPT2 in autism has so far been inconsistent, with studies showing evidence for both (Hobson, 1984; Leslie and Frith, 1988; Tan and Harris, 1991; Yirmiya et al., 1994; Leekam et al., 1997; Warreyn et al., 2005; Hamilton et al., 2009). Here we will examine studies and the methods they have used, taking into account what they add to the study of VPT in autism.

## Inclusion criteria

An exhaustive search of the literature on VPT in autism was conducted using PubMed, web of science and Google Scholar. The search terms entered were “autism”/“ASD” and “visual perspective taking”/“VPT.” Thirteen papers were identified which appeared to fit these criteria. All 13 papers examining VPT in autism have been included in this review.

Though studies aim to examine either VPT1 *or* VPT2, many of the tasks that have been used to test VPT could be completed using either, i.e., some VPT2 tasks could be completed using a simple line of sight VPT1 strategy. Here we discuss all studies which have examined VPT in autism and evaluate whether they fall into the category of VPT1 or VPT2.

## VPT in autism

VPT has often been examined using tasks which ask questions about item visibility (Moll and Tomasello, 2004). In these studies, the child is presented with an item which is either in view or occluded from an adult. The child has to respond to whether the adult can see the item. Explicit studies of item visibility in typically developing (TD) children have shown that they are able to respond accurately from around 2 years old (Moll and Tomasello, 2004, 2006). Hobson (1984) examined VPT in adolescents with autism and VMA (verbal mental age) matched TD children using a “hide and seek” game paradigm, and found that the ability to perform VPT was intact. Participants were presented with a display which included hiding holes and two figures. The participant had to “hide” their figure from the other, indicating in which hole the figure would need to be placed so that they would not be seen. The participants with autism performed similarly to the ability matched TD children. These results have since been replicated using a similar hiding paradigm (Reed and Peterson, 1990; Tan and Harris, 1991; Reed, 2002). The findings from these studies suggest that children with ASD are able to understand the concept of “hiding” and what other people can see.

VPT has also been examined using line of sight paradigms. Leslie and Frith (1988) used a line of sight paradigm to investigate VPT in children with autism. Participants were presented with a scene in which a doll sat on one side of a cardboard screen and a counter was placed on the same side as the doll, or the opposite side. The child had to respond to whether the doll could see the counter. All of the autistic children were able to complete the task, suggesting that they had a basic understanding of what the doll could and could not see.

Baron-Cohen (1989) used a line of sight paradigm to examine VPT in children with autism and a group of TD children. Children were presented with a task in which an experimenter would orient their gaze or body toward one of six items surrounding the child and the child would have to identify which item the experimenter was looking to. The results showed that 92.5% of the children with ASD passed the task compared to 94.4% of TD children, suggesting VPT to be intact in the ASD group. Baron-Cohen's study has been replicated since, though findings have not been quite as clear. Leekam et al. (1997) compared a group of ASD children to a group of VMA matched typical children on Baron-Cohen's perspective taking task. Though results showed no significant difference between the groups, there was a ceiling effect in the TD group (100%) whereas the ASD group scored on average much lower (66.6%). They also found that VMA was a significant predictor of performance, with those of lower VMA showing more difficulty with the task.

Warreyn et al. (2005) also conducted a replication of Baron-Cohen (1989) and found that young children with autism performed worse on the VPT task compared to age matched TD children. Similarly to Leekam et al. (1997), they found VMA to be a significant predictor of VPT ability. The authors suggested that VPT may develop later in children with autism and that they may be delayed compared to TD children.

All of the studies presented above (Hobson, 1984; Leslie and Frith, 1988; Baron-Cohen, 1989; Reed and Peterson, 1990; Tan and Harris, 1991; Leekam et al., 1997; Reed, 2002; Warreyn et al., 2005) can be classified as Level 1 VPT tasks on the basis that they examine line of sight.

VPT has also been examined using questions about item appearance. Mizuno et al. (2011) used a paradigm similar to that of Masangkay et al. (1974), in which adults with autism were shown a picture card with two sides. Participants were asked to identify which side they would see or another person would see in two different VPT conditions. In the first condition participants were asked a “what” question (“*what can I see?*” or “*what can Sarah see?*” vs. “*What can you see?*”). In the second condition they were asked a “who” question (i.e., “*who will see the carrot?*”). Results showed that participants with autism were slower in the “what” condition than in the “who” condition. The authors argued that this was a result of difficulty switching between personal pronouns (“what can *you* see?” requires the participant to make the link between “you” being themselves'), which people with autism often find difficult (Lee et al., 1994). As the study uses a classic VPT1 paradigm, it seems most appropriate to label this a VPT1 task.

Hobson (1984) compared children with autism to a group of younger, VMA matched typical children. To examine VPT, Hobson used an object appearance task in which children had to identify the viewpoint of a third person (a doll). Typical and ASD children were presented with a cube which had a different color on each vertical face. The child was given a chance to familiarize themselves with the cube. Once familiarized the experimenter would place a doll (Fred) at one side of the cube and ask “*Fred sits here, which colour can he see?*” or “*place Fred so he can see the ___*.” The child was then given a second doll (Mary) and asked *to* “*put Mary so that Mary sees the same as Fred sees*.” Results showed that there was no significant effect of group, with the ASD children performing similarly to the typical children. Hobson did find a significant effect of verbal ability in the ASD group, with higher functioning ASD children performing better. This is consistent with the findings from Warreyn et al. (2005) and (Leekam et al., 1997), and suggests that verbal ability may be an important predictor of VPT. It is also worth noting that neither group performed at ceiling level in Hobson's task meaning any group differences should be clear. As the task could be completed using a VPT1 strategy in which participants use line of sight to respond rather than performing a first person transformation it seems appropriate to define this as a level one VPT task.

Reed and Peterson (1990) also examined VPT in children with autism alongside ToM using an item appearance paradigm. Thirteen ASD children and 13 VMA matched TD children were tested on their ability to rotate a familiar item (a toy) so that the experimenter could see a distinct feature (i.e., “*turn it so that I can see the nose*”). Four different toys were presented and children had to score 100% across all four trials to pass. In contrast the cognitive perspective taking task required the children to perform the Sally-Anne ToM task (Baron-Cohen et al., 1985). The authors found that the children with autism performed similarly to the typical children in the VPT task, but worse in the cognitive perspective taking task. The authors concluded that it could not be the social aspect of ToM that participants with autism had difficulty with, as the VPT task was also social and that poor ToM may be a result of impaired abstract thinking. These findings suggested that VPT and mentalizing are dissociable abilities, with VPT tapping into a different process then ToM. However, the authors found a ceiling effect amongst both the typical and autistic children in the VPT task. This makes it possible that group differences may have been masked due to the task being particularly easy for both groups of participants. This task was classified as a VPT2 task by the authors on the basis that it meets criteria for two people viewing an object from different vantage points (Flavell et al., 1981). However, participants could also use a basic line of sight (VPT1) strategy (turning the item until the feature (i.e., nose) was in the line of sight of the viewer) to respond. The distinction between level one and two VPT are blurred in this task, and it may be more appropriate to label this a VPT1 task.

Tan and Harris (1991) examined VPT in children with autism using an item location task. Twenty children with autism and 20 VMA matched TD children were tested on their ability to identify the view one of two soft dolls would have of a third object (i.e., *which object would John say was* “*in front?*”). The authors also measured the children's ToM using a desire understanding task, presenting the children with scenarios in which someone was offered food that they did or did not like. Children had to respond to whether the person would be happy or unhappy with the offer. There was no significant effect of group on either task, with the autistic children performing similarly to the typical children on both VPT and desire understanding. As with Reed and Peterson's task, Tan and Harris also found a ceiling effect across both groups of participants which may have masked any group differences. The authors concluded that a global social deficit in autism is unlikely, and that impairment may be related to process and task specific delays. As this task measures how two people seeing a given object may view it differently due to a change in orientation or location (i.e., for Mary, the pencil is in front of the block, whereas for John the pencil is behind the block) it can be considered a VPT2 task.

Yirmiya et al. (1994) examined VPT in children with ASD using an object rotation paradigm in which children were presented with familiar item (toys) on a rotating table. The task required both object rotation and item appearance (“how would this look to me”). ASD children were compared to age and IQ matched TD children on their ability to turn a turntable containing 3 or 10 items so that it matched the point of view of the experimenter. Children were instructed to “turn it around so that you will see it from where you are in the same way that I see it from where I am” or “turn it around until you see it in the exact same way that I see it now from where I am standing.” They found that children with ASD showed a higher number of errors than the typical children. Errors were further categorized into two different types: incorrect (in which the answer was simply wrong) or egocentric (in which the child displayed the turntable with their own point of view). Children with autism were found to display more incorrect errors in the 10 item trials, and more egocentric errors in the 3 item trials. This suggests that the 10 item trials were more reliant on memory, as if both trial types were equated for difficulty you would expect to see similar types of errors across both. This task demands the calculation of two different viewpoints and is clearly a VPT2 task, but as the authors note it has heavy memory demands which may limit performance.

Hamilton et al. (2009) used a related paradigm to examine VPT, mental rotation and ToM ability in a group of ASD children compared to verbal ability matched TD children. Two further groups of TD children were also included in the study, a typical mid-age range group and a typical older group. For the VPT task children were presented with the toy on the turntable and asked to identify their own point of view on the answer sheet. The toy was then covered and a doll placed at another spot on the table. The child was asked to identify the view of the toy the doll would have when the pot was lifted. For the mental rotation task children were shown a toy on a turntable and asked to identify which picture on their answer sheet matched their view. The toy was then covered and rotated and the child asked to identify which view they would see when the pot was lifted. ToM was assessed using a battery of different ToM tasks, including diverse desires and the Sally-Anne task (Baron-Cohen et al., 1985). Results showed that the children with ASD were significantly worse on the VPT trials compared to the typical children, but performed better on the mental rotation task. It was also found that VPT was significantly predicted by ToM score, suggesting mentalizing is important for perspective taking. The authors suggested that VPT relies on the same cognitive systems as ToM. This is the only study reviewed which includes both a social and non-social spatial task, as well as a measure of ToM. The task attempts to integrate different task demands (viewer and item rotation, item appearance questions) making it possible to start pinpointing specific difficulties with VPT. The use of a control spatial (non-social) task also allows the authors to make clear conclusions about which aspects of VPT that people with autism find difficult (social as opposed to the spatial). We suggest that as the task explicitly requires participants to say what one object would look like from two different points of view, with no line of sight information available (the target was covered with a pot), that this be classified as a VPT2 task.

Dawson and Fernald (1987) also examined VPT in children with autism using an object rotation paradigm in which children had to orient an item a certain way for the experimenter to see it. No control group was included in the study. Participants were presented with cards, blocks and various picture and asked to orient it “*so the experimenter could see the face/tail etc ….*” None of the children scored at ceiling level on the task, and performance correlated with social skills, but without a control group it is hard to interpret this data.

David and colleagues examined VPT and ToM in high functioning adults with Asperger syndrome compared to age and IQ matched TD adults. Participants completed two tasks, one examined VPT and the other examined ToM. In the ToM task participants were presented with a virtual image of a person with one item either side of them. The person could be displaying one of three possible body, face and hand postures (positive, neutral, or negative) toward one of the objects. An example of a positive hand gesture would be pointing, whereas negative would be holding the hand out with the palm facing forwards (similar to a “stop” signal). The participant's task was to identify which object the other person desired (mentalizing for other) or which they would desire themselves (mentalizing for self). In the VPT task the participant was presented with the same image of the person with two objects, one of which was elevated. The participant had to identify which object was elevated from their own point of view, or from that of the other person using a laterality judgment (i.e., the item on *my left* is higher). Measures of speed and accuracy were taken from each participant. In the ToM task results showed that the ASD participants were significantly slower and less accurate at identifying the correct answer when mentalizing for other. They were also trending toward slower mentalizing for self (as accuracy on this task was subjective accuracy could not be measured). There were no differences found between groups for speed or accuracy in the VPT task, for self or other. The authors acknowledged that the VPT task may have been too easy compared to the mentalizing task which may explain differences across tasks. One limitation is that this task does not require participants to take the visual perspective of the other, but only to judge what is on the left or right. Spatial-transformation tasks (Parsons, 1987; Zacks et al., 1999) requires participants to make laterality judgments about an item in relation to another person, but it is not clear if these are the same as VPT tasks. Further research is needed into these paradigms in order to assess where they fall in relation to perspective taking.

Similarly, Zwickel et al. (2011) examined VPT and ToM in adults with autism and age and IQ matched TD adults using a laterality judgment paradigm. In the VPT task participants viewed videos of animated triangles (Castelli et al., 2002), and during the videos a dot appeared to the left or right of the triangle. Participants were asked simply “*was the dot on your left or right*.” On incongruent trials a dot on the participant's left fell on the right of the triangle (or vice versa), while on congruent trials a dot on the participant's left was also on the left of the triangle (or both on the right). Critically, this congruency only arises if the triangle is perceived as an animate active creature. Both typical and autistic participants showed a congruency effect in this task, demonstrating that they could spontaneously consider the left/right orientation of an animated shape. However, the autistic participants were less good at judging the mental states of the triangles in the same animations. This is consistent with the findings of David et al. (2010). Similarly, it is not clear if this task truly demands calculation of the *visual perspective* of another agent rather than just their orientation. More research is needed to explore the use of visuo-spatial perspective taking paradigms in autism.

## Evaluating VPT in autism

We have reviewed 13 studies of VPT in autism, and suggest that 7 of these assessed VPT1, 3 assessed VPT2 and 3 were unclear or assessed laterality (see Table 1). Of the 7 studies examining VPT1, 5 report no differences between typical and autistic participants while the other 2 find that participants with autism perform worse than typical participants. Of the 3 studies examining VPT2, 2 report group differences and the third does not.

**Table 1:** **Summary of studies included in this review**.

	**Participants with ASD**			**Typical participants**			**Experimental tasks**			
**Study**	**Number**	**Mean age**	**Mental age**	**Number**	**Mean age**	**Mental age**	**IQ measure**	**Task**	**Level**	**Result**
Yirmiya et al. (1994)	18	12.5	12.3	14	12.0	12.5	WISC	Turn turntables to match my point of view	2	Sig effect of group (TD > AS)
Hamilton et al. (2009)	23	8.0	4.4	23	4.2	4.8	BPVS	Which bear will Susan see'	2	Sig effect of group (TD > AS) on VPT, AS better at MR
Reed and Peterson (1990)	13	12.0	7.1	13	7.1	–	WISC/ WAIS	Turn the turn table so I see the	1	No sig difference between groups
Hobson (1984)	12	13.8		27	5.7	–	WISC	What can “…” see?	1	No sig difference between groups
Tan and Harris (1991)	20	12.8	7.7	20	6.1	6.5	PPVT	Which item is in front of…	2	No sig difference between groups
David et al. (2010)	19	36.4	>30	15	31.2	>30	WAIS	Which object is elevated from your/their perspective?	lat	No sig difference between groups
Zwickel et al. (2011)	19	37.0	>30	18	39.0	>30	WAIS	Which side of the triangle is the dot?	lat	Sig diff on WRONG responses only
Warreyn et al. (2005)	20		4.3	20	5.6	4.7	RDLS	What am I looking at?	1	Sig effect of group (TD > AS)
Reed (2002)	25	12.0	8.2	25	6.8	7.11	PPVT	Can you hide Susie where no-one else can see	1	Sig effect of group (TD > AS)
Leekam et al. (1997)	12	11.8	5.4	12	5.8	–	TROG	Which toy am I looking at	1	No sig effect of group, but trending toward TD > AS
Baron-Cohen (1989)	20	11.1	5.5	27	4.5	–	BPVS	Which toy am I looking at	1	No sig difference between groups
Leslie and Frith (1988)	18	13.1	7.2	12	8.8	6.9	BPVS	Can the doll see the counter	1	No sig difference between groups
Dawson and Fernald (1987)	16	11.1	5.8	–	–	–	WISC and others	Turn it so I can see the …	mix	VPT sig related to social skills

There are several interesting issues arising from this review which can guide future research. One important problem is that the boundary between VPT levels one and two is not always clear. A task might be intended to assess VPT1, but participants might choose to use a VPT2 strategy. Or if a study designed to measure VPT2 could also be completed using line of sight, it is possible that people with autism could pass based on this information. This is particularly the case in studies which name the item which can be seen from a particular location [e.g., *place Fred so he can see the red side*, (Hobson, 1984)]. Here the child need only consider Fred's line of sight to the red part of the cube, but some children might prefer to consider the relationship of the whole cube to the rest of the scene including the child's own viewpoint. Thus, this task could be solved by a VPT1 or VPT2 strategy. To minimize this issue, we suggest that line of sight tasks seem to be the clearest way to assess VPT 1 (Leslie and Frith, 1988; Baron-Cohen, 1989; Leekam et al., 1997; Warreyn et al., 2005), whilst item appearance tasks appear to be the best way to assess VPT2 (Hamilton et al., 2009) see Figures 1A,B.

A related issue is the use of different strategies by different participants. However well an experimenter designs a task, it is always possible that participants could solve the puzzle in a different way. For example, many VPT tasks could potentially be solved with a purely spatial mental rotation (Zacks and Tversky, 2005). This approach is less efficient, but it is possible that different groups of participants prefer to use different strategies. One way to approach this issue is to consider the use of appropriate control tasks to assess other cognitive skills such as children's memory abilities (especially for complex displays), their language skills (for complex questions) and their abilities to perform spatial transformations. The comparison of an experimental task and a closely matched control task in the method of fine cuts (Frith and Happe, 1994) would allow for close examination of the cognitive components which distinguish the different levels of perspective taking. For example, Surtees et al. (2013) suggests that VPT2 requires an embodied spatial transformation while VPT1 does not. If this is the case, then VPT2 abilities should correlate with performance on other tasks requiring embodiment, but not to mental rotation tasks that do not involve bodies. If different groups of participants use different strategies to perform VPT tasks, this might also emerge in the relationship between their VPT skill and other cognitive skills.

Furthermore, this raises another important question concerning how the social and spatial elements of VPT2 fit together: Does intact VPT require spatial *and* social information, or could it be done using just one of these? If VPT2 can be completed using social *or* spatial information it makes sense that it can be unimpaired even in the face of significant ToM deficits, as participants' could rely on the use of spatial information to complete a task. Langdon and Coltheart (2001) suggested that tasks using questions about item location (i.e., Tan and Harris (1991)) were particularly open to completion via spatial cues making it possible for those with social difficulty to perform. However, if VPT2 requires the integration of both spatial and social information to be effective, then even good spatial ability would not completely compensate for poor social processing. Again, determining the strategies and cognitive mechanisms that different participants use to perform VPT tasks is critical here.

Another issue concerns the participant populations tested. The majority of studies presented in this review were conducted on children, and several on groups of children with impaired cognitive functioning. It is difficult to collect reaction time data from children, meaning that more subtle differences in VPT ability related to an inability to integrate social and spatial information may be missed. The two studies conducted with adults (David et al., 2010; Zwickel et al., 2011) did not find group differences but did not use typical VPT tasks. It is possible that [as found in ToM research (Ozonoff et al., 1991)], high functioning adults may be able to pass VPT. Whether this is due to a better understanding of the questions asked, or the development of an alternative strategy for completion of the tasks is unclear. Both of these suggestions warrant further research and careful consideration of the paradigms used to examine VPT.

There are also issues in the lack of consistency in matching groups. Though some of the studies have used rigorous matching techniques (Yirmiya et al., 1994; Hamilton et al., 2009; David et al., 2010), others took no measure of cognitive ability in their typical participants. Both Reed and Peterson (1990) and Hobson (1984) argue for evidence of unimpaired VPT2 performance in autism. However, they both compared groups of older ASD children to younger typical children. This suggests that at the very least the participants with autism may be displaying a delay in the development of VPT (similar performance to younger children as opposed to an age matched group) and that it may be inappropriate to label their performance as unimpaired. By comparing ASD participants to both age and ability matched control participants, it becomes possible to make stronger claims as to whether performance on a task is normal, impaired or simply delayed. These findings present a strong case for using carefully chosen control groups in studies looking for evidence of impairment in a population such as autism.

## Future directions

Understanding the relationship between VPT and ToM is important. Both of these require the consideration that the other person has a different representation of the world to oneself, either a different visual representation or a different belief. Early studies suggested that VPT is intact in autism while ToM is impaired. This motivated the idea that it is easy to distinguish visual representations of self and other because VPT allows concrete feedback by physically moving to a different location (Leslie (1987). In contrast, ToM requires more abstract representations which people with ASD find difficult. More recent data suggest that VPT2 and ToM are linked in typical children (Hamilton et al., 2009), in those with specific language impairment (Farrant et al., 2006) and in the brain (Aichhorn et al., 2006). This implies that VPT2 and ToM may share similar underlying cognitive mechanisms. Certainly, many false belief tasks rely on the ability to distinguish what people have seen (Sally did not see Anne move the marble) which draws upon VPT. VPT has been found to activate the temporo-parietal junction, an area commonly found to be activated by ToM tasks (Aichhorn et al., 2006). It has been suggested that ToM may be driven by different mechanisms or strategies in people with autism compared to TD people (Tan and Harris, 1991). We believe it may also be worth considering that this could also be the case for VPT in people with autism. If both are being driven by different mechanisms it may explain why some studies have shown VPT to be unimpaired alongside impaired ToM (Tan and Harris, 1991) and vice versa (Hamilton et al., 2009). Further studies of the relationship between ToM and VPT would be very useful, as would studies examining the cognitive mechanisms which underlie each.

It is also worth considering how researchers can tease apart the specific contributions of social and spatial mechanism in VPT. Several VPT tasks have been used successfully in TD individuals which have allowed researchers to emphasize the spatial or social aspects. The use of these paradigms may provide us with useful information about perspective taking in autism. As described earlier, Samson et al. (2005) investigated the social components of VPT in an implicit perspective taking task. Results showed that participants could not ignore the perspective of an avatar, and made slower responses when the avatar could not see something which the participant could see. Another VPT task with strong social demands is Keysar et al. (2003) director task. In this task the participants stand behind a shelf holding several items while another person stands in from (the director) and gives instructions of which items to choose. Not all items are visible to the director and so the participant must be able to take the directors perspective into account to avoid choosing items that they cannot see. The authors found that participants were not able to inhibit their own perspective when choosing items and often made incorrect responses. This task has been argued to have a strong ToM component as it relies on the ability to represent someone else's false belief (the director believes the “big jar” is the one they can see, but there is a bigger jar on view to the participant). Both of these tasks would provide interesting ways of measuring the social components of VPT.

Kessler and Thomson (2009) developed a task in which they were able to examine the underlying spatial components present in perspective taking (termed spatial perspective taking, or SPT). Participants were presented with images of a human avatar seated at a table with an item to either side of them (a flower and a gun). The position of the avatar at the table was rotated to be more or less congruent with the position of the participant (providing changes in the angular disparity between the avatar and viewer). Participants had to make laterality judgments in regards to the placements of the items from the avatars viewpoint. The authors found that the larger the angular disparity between then avatar and the viewer, the longer participants took to respond. This demonstrated the underlying spatial transformation that the participant completed in order to put themselves in the place of the avatar, highlighting the importance of spatial mechanisms in perspective taking. These findings show that in order to take a first person perspective, an embodied transformation (where the viewer transforms their body to match that of the avatar or target viewpoint) is often necessary. Mazzarella et al. (2013) built upon these findings, investigating the neural underpinnings of spatial perspective taking with a similar experiment which examined SPT under fMRI. In this task participants were scanned whilst making egocentric (what item is on your left) vs. altercentric (which item is on their left) judgments about the placement of items on a table [using the same paradigm as Kessler and Thomson (2009)]. This was designed to tease apart the differences in transforming the self to a different position vs. transforming the self into someone else's position. The authors found that though both types of transformation show similar behavioral data patterns, there was a neural distinction between the areas engaged during egocentric and altercentric perspective taking. This suggests that multiple strategies may be used for putting the self in a different place. These tasks both provide clear mechanisms for teasing out the spatial components of VPT, as well as interesting avenues to explore in people with autism.

Recently, researchers have also begun to examine the link between autistic traits in TD individuals and how this affects perspective taking. As VPT is a sociocognitive ability which impacts on social interaction, it stands to reason that those with poorer social skills might also show poorer perspective taking ability. Three studies have examined this question. First, Kessler and Wang (2012) examined participants using the same task from Kessler and Thomson (2009). A measure of autistic traits in these participants was taken using the Autism Quotient (AQ, Baron-Cohen et al., 2001). The authors found that participants who scored higher on the AQ (Baron-Cohen et al., 2001) showed more difficulty with performing egocentric transformations in a VPT2 task than low AQ scorers. However these high AQ participants also showed quicker response times. The results further suggest that using an embodied perspective slowed participant's responses at higher angular disparities as it took longer for the participant to transform their body to match that of the target. Participants with poorer embodiment skills did not slow their response times, most likely due to the use of a non-embodied transformation strategy. Using a very similar task, Brunye et al. (2012) also found that high AQ scorers had difficulty with using an egocentric reference frame in VPT2, though in this study these participants showed slower response times. This suggested that these high AQ participants were attempting to use an embodied strategy, but found it more difficult. Finally, Shelton et al. (2012) also found a link between spatial skills and autistic traits. In their study participants were presented with a three mountains (Piaget and Inhelder, 1956) like scene, in which an array of three buildings were visible to participants. A doll was placed facing the array and participants had to respond to which point of view the doll would see. Their study showed that participants with high AQ scores were less accurate than those with low AQ scores.

Together, all these suggest that autistic traits (as well as autism itself) can influence a participant's ability to perform VPT2. These studies are important for two reasons. Firstly they demonstrate that autistic traits (not just a diagnosis of autism) impact on the ability to take another perspective. Secondly, they add weight to the argument that those who find it difficult to complete VPT2 using an embodied perspective may develop an alternative strategy. The findings from these studies provide a strong motivation for considering the types of participant samples used in VPT2 research and measuring traits which could affect performance alongside carefully designed paradigms and tasks.

## Conclusions

From the evidence presented in this review, the majority of studies suggest that whilst VPT1 may be intact in people with autism, VPT2 is impaired. We suggest that this is a result of the cognitive mechanisms involved in the different levels of VPT, with VPT2 drawing on embodied spatial transformations and triadic representations (Surtees et al., 2013) more than VPT1. Future studies should carefully consider the cognitive differences between VPT1 and VPT2. Furthermore, there is evidence to suggest that the ability to perform egocentric transformations (a process which can be seen as the first step in completing VPT2 (Yu and Zacks, 2010) could be impaired in autism, but may also be affected in people with high levels of autistic traits (Brunye et al., 2012; Kessler and Wang, 2012; Shelton et al., 2012). It is clear that more research is needed into the processes related to VPT2 in autism in order to clarify these suggestions. There is a strong case to be made for more inclusion of measures of general spatial ability in studies on VPT and the use of a “fine cuts” technique when designing studies. This will allow researchers to tease apart impairments in the spatial demands of a task vs. the social. The recommendations set out in this review provide a strong motivation for investigating VPT in autism and shed light on why findings so far are inconsistent.

### Conflict of interest statement

The authors declare that the research was conducted in the absence of any commercial or financial relationships that could be construed as a potential conflict of interest.
